# Development of a nomogram prediction model for depression in patients with systemic lupus erythematosus

**DOI:** 10.3389/fpsyg.2022.951431

**Published:** 2022-09-14

**Authors:** Haoyang Chen, Hengmei Cui, Yaqin Geng, Tiantian Jin, Songsong Shi, Yunyun Li, Xin Chen, Biyu Shen

**Affiliations:** ^1^Department of Nursing, Shanghai Children’s Medical Center Affiliated to Shanghai Jiao Tong University School of Medicine, Shanghai, China; ^2^Department of Nursing, Nantong Second People’s Hospital, Nantong, China; ^3^Department of Rheumatology, The Second People’s Hospital of Changzhou, Changzhou, China; ^4^Department of Nursing, The Second Affiliated Hospital of Nantong University, Nantong, China; ^5^School of Nursing, Shanghai Jiao Tong University School of Medicine, Shanghai, China

**Keywords:** systemic lupus erythematosus, depression, nomogram, prediction, China

## Abstract

Systemic lupus erythematosus (SLE) is an inflammatory autoimmune disease with depression as one of its most common symptoms. The aim of this study is to establish a nomogram prediction model to assess the occurrence of depression in patients with SLE. Based on the Hospital Anxiety and Depression Scale cutoff of 8, 341 patients with SLE, recruited between June 2017 and December 2019, were divided into depressive and non-depressive groups. Data on socio-demographic characteristics, medical history, sociopsychological factors, and other risk factors were collected. Between-group differences in clinical characteristics were assessed with depression as the dependent variable and the variables selected by logistic multiple regression as predictors. The model was established using R language. Marital status, education, social support, coping, and anxiety predicted depression (*p* < 0.05). The nomogram prediction model showed that the risk rate was from 0.01 to 0.80, and the receiver operating characteristic curve analysis showed that the area under the curve was 0.891 (*p* < 0.001). The calibration curve can intuitively show that the probability of depression predicted by the nomogram model is consistent with the actual comparison. The designed nomogram provides a highly predictive assessment of depression in patients with SLE, facilitating more comprehensive depression evaluation in usual clinical care.

## Introduction

Systemic lupus erythematosus (SLE) is a chronic autoimmune disorder that contributes to limitations in physical, psychological, and social functioning. Many cross-sectional studies have found a high prevalence of depression among persons with autoimmune diseases. In 1999, the American College of Rheumatology *Ad Hoc* Committee on Neuropsychiatric Lupus published standardized nomenclature and diagnostic recommendations for the 19 syndromes of neuropsychiatric SLE. Based on the Diagnostic and Statistical Manual of Mental Disorders Fourth Edition criteria, five of these 19 syndromes are psychiatric: depression, anxiety disorders, cognitive dysfunction, psychosis, and acute confusional state (American College of Rheumatology, 1999). Among these psychiatric manifestations, depression and anxiety disorders represent the most common complaints (Nikpour et al., 2014). Further, depression has been associated with greater disease severity in populations with autoimmune diseases (Shakeri et al., 2015). Increased immune activation associated with depression is a plausible biological pathway that could trigger autoimmune disease (Deverts et al., 2010).

Specific to SLE, the prevalence of depression has been reported to be twice that in the general population (Bachen et al., 2009). At the same time, a history of depression has been associated with 2.5 times greater risk of developing SLE as compared with the absence of a history of depression (Roberts et al., 2018). Depression entails “feelings of sadness, despair, emptiness, discouragement, or hopelessness; having no feelings; or appearing tearful” (American Psychiatric Association, 2013). In patients with SLE, depression can have a profound impact on health and wellbeing; this includes an increased incidence of cardiovascular diseases, myocardial infarction, suicidal ideation, and physical disability; decreased quality of life; and a higher risk of premature mortality (Zhang et al., 2017). Depression-like symptoms in patients with autoimmune diseases results in poor disease control; depression is generally thought to be associated with increased disease activity, decreased medication adherence, and decreased work productivity. Up to 50% of patients with autoimmune diseases show impaired health-related quality of life and exhibit depression-like symptoms. The immune system not only leads to inflammation in affected organs but also mediates behavior abnormalities, including fatigue and depression-like symptoms (Pryce and Fontana, 2017). Physical exercise and psychological interventions can act as adjuncts to traditional medical therapy for improvement in depression and quality of life for SLE (Fangtham et al., 2019).

Therefore, early identification and intervention for depression not only reduces patients’ psychological burden but also improves their quality of life. While stress, including psychosocial factors (inclusive of having SLE itself), is a relevant factor, depression can also be caused by organic changes in the brain (neuropsychiatric SLE) and therapeutic agents such as steroids (side effect). To develop a depression assessment tool specific to patients with SLE, all the above seem to be essential elements (Palagini et al., 2013).

Estimates of depression vary by diagnostic and screening tools (Zhang et al., 2017). However, in addition to patient psychology, socio-demographic characteristics can also be predictive of depression. Patients with lupus who have depression will deliberately conceal their inner suffering. Other people are only alerted to their condition when it develops to a serious level, such as with self-harm and suicidal tendencies; thus, the best time for intervention passes. Therefore, looking for simple and efficient methods to screen high-risk patients for depression is necessary. In this context, nomograms—statistical models for individualized predictions of clinical events—have been developed to provide risk estimates for the presence of a disease or event during the future course of the disease (Jia et al., 2019). Nomograms can be used to perform single-item scoring and aggregate total scores for the corresponding variables of each influencing factor in Cox or logistic regression models and predict the corresponding probability based on the total score. This study is an attempt to establish a predictive nomogram for depression based on socio-demographic characteristics, medical history, sociopsychological factors, and other risk factors in patients with SLE. Through this study, we seek to establish a comprehensive visual predictive model for depression in patients with SLE. The proposed nomogram can inform clinical decision-making and enable the identification of individuals at high risk for depression, who should undergo further screening tests, thus promoting early detection of and intervention for depression.

## Materials and methods

### Participants

Patients with SLE were recruited from a hospital in Nantong between June 2017 and December 2019. In total, 360 consecutive patients with SLE were invited to participate in this cross-sectional study. All patients fulfilled the 1997 American College of Rheumatology revised criteria for the classification of SLE. Patients were excluded if they (1) did not complete the questionnaire or (2) had comorbidities (e.g., serious infections or cardiac, respiratory, gastrointestinal, neurological, or endocrine diseases) that could influence SLE activity. The Hospital Ethics Committee approved this study (2017-016) and written informed consent was obtained from all participants.

### Demographic and clinical characteristics

Demographic data included gender, age, body mass index, residential area, marital status, education (years), employment status, income, medicare, smoking, alcohol consumption, and exercise. Clinical data, obtained through medical records and patients’ self-reports, included disease duration (years).

### Measures

#### Systemic Lupus Erythematosus Disease Activity Index

The SLE Disease Activity Index (SLEDAI; Hochberg, 1997) was used to measure disease activity over the past month. It evaluated 16 clinical characteristics and eight laboratory indicators. Higher scores indicated more severe disease activity.

#### Hospital Anxiety and Depression Scale

The Hospital Anxiety and Depression Scale (HADS) (Bjelland et al., 2002) was used to estimate levels of anxiety and depression. It consists of 14 items, with seven items each for anxiety and depression. The cutoff for the identification of the occurrence of anxiety and depression was 8 points, and higher scores indicated more severe mood disorders. The HADS demonstrated good validity and reliability among the Chinese patient population (Zhang et al., 2017).

#### Pittsburgh Sleep Quality Index

The Pittsburgh Sleep Quality Index (PSQI) (Tsai et al., 2005) was used to estimate the quality of sleep over the past month. The PSQI contains a total of 24 questions (19 self-rated questions and five rated by the bed partner or roommate), and 18 items divided into seven domains of sleep difficulties including sleep quality, sleep onset latency, sleep duration, habitual sleep efficiency, sleep disturbance, use of sleep medicine, and daytime dysfunction. The sum of each domain generates the overall index for sleep quality evaluation. The total score ranges from 0 to 21, with higher scores indicating poorer sleep quality. The PSQI demonstrated good validity and reliability among the Chinese patient population (Chen et al., 2022).

#### Coping style questionnaire

Xie’s (1998) 20-item Coping Questionnaire was used to estimate coping style. On this self-assessment scale consisting of positive and negative responses, each item is rated on a four-point Likert-type scale the score range is from 0 to 3; coping tendency greater than 0 is a positive attitude, less than 0 is a negative attitude. This scale demonstrated good validity and reliability among the Chinese patient population (Sun et al., 2019).

#### Social Support Rating Scale

The Social Support Rating Scale (SSRS) (Piel et al., 2017) was used to estimate levels of social support. It consists of three subscales (subjective support, objective support, and support availability). Each item is scored on a five-point Likert scale from 0 to 4. The total score, which is the sum of the scores of subjective support, objective support, and support availability, is used as a measure of the current total social support status, with higher scores indicating more social support. The SSRS demonstrated good validity and reliability among the Chinese patient population (Xu et al., 2019).

### Statistical analysis

SPSS 24.0 (IBM Corp., Armonk, NY, United States) and R 3.5.2 software were used to process the data. According to the data and distribution characteristics, the two-sample *t*-test, Wilcoxon rank sum test, and χ^2^ test were used to compare the differences between the depression and non-depression groups; logistic regression was used to analyze the influencing factors of depression. Depression was the dependent variable, and the variables selected by logistic regression were used as predictors. The nomogram prediction model was established using R language. The predictive and prognostic nomograms were developed using the “rms” package in R software based on the independent predictive factors. Additionally, the area under the receiver operating characteristic curve (AUC) was used to evaluate the discrimination of nomograms. The AUC value ranges between 0.5 and 1; the higher the value, the higher the resolution of the nomogram. Moreover, calibration curves were established for the nomogram. The significance level was set at α = 0.05.

## Results

Nineteen patients with SLE did not complete the questionnaire; thus, the data of 341 patients were analyzed. The baseline demographic characteristics of patients with and without depression are summarized in Table 1. The average age in the depression group was 35.6 ± 10.89 years. Eighty-nine cases (two men, 87 women) fulfilled the criteria for depression, and the cumulative incidence of depression was 26.1%. There were significant differences in residential area, marital status, education, and income between the depressive and non-depressive groups. Regarding the analysis of sociopsychological factors, anxiety, SSRS, PSQI, and coping style scores were significantly different (*p* < 0.01) between the depression and non-depression groups.

**TABLE 1:** Baseline characteristics of patients with and without depression.

Characteristic	Depression (*n* = 89)	Non-depression (*n* = 252)	*P*-value
**Socio-demographic characteristics (%)**			
**Gender**			0.73
Male	2 (2.24)	9 (3.57)	
Female	87 (97.76)	243 (96.43)	
**Age, mean (SD), y**	35.6 (10.89)	37.33 (11.85)	0.11
**Residential area**			0.004**
City	27 (30.33)	123 (48.80)	
Village	62 (69.67)	129 (51.20)	
**Marital status**			<0.001***
Married	84 (94.38)	185 (73.41)	
Unmarried	5 (5.62)	67 (26.59)	
**Education, y**			<0.001**
≤9	59 (66.29)	95 (35.79)	
>9	30 (33.71)	157 (64.21)	
**Employment status**			0.42
Employed	43 (48.31)	141 (59.95)	
Unemployed	45 (51.69)	111 (40.05)	
**Income**			0.02*
Low	25 (28.09)	84 (33.33)	
Medium	47 (52.80)	92 (36.50)	
High	17 (19.10)	76 (30.15)	
**Medicare**			0.15
Yes	50 (56.17)	167 (66.26)	
No	39 (43.83)	85 (33.74)	
**Exercise**			1.00
Yes	38 (42.69)	106 (42.06)	
No	51 (57.31)	146 (57.94)	
BMI (SD)	22.75 (3.71)	22.31 (3.91)	0.811
**Medical history (*n*, %)**			
Smoker	0 (0)	6 (2.38)	0.35
Alcohol drinker	0 (0)	11 (4.36)	0.07
Course of disease (SD)	7.69 (6.62)	7.36 (6.43)	0.68
SLEDAI (SD)	4.72 (3.58)	4.3 (3.47)	0.344
**Socio-psychological factors, mean (SD)**			
Anxiety (%)	72 (81.00)	50 (19.84)	<0.001***
SSRS	39.70 (5.68)	42.49 (6.74)	0.001**
PSQI	6.39 (3.15)	4.37 (2.86)	<0.001***
Coping (%)			<0.001***
Positive	72 (80.89)	110 (43.65)	
Avoidance or giving up	17 (19.11)	142 (56.35)	

SD, standard deviation; BMI, body mass index; SLEDAI, Systemic Lupus Erythematosus Disease Activity Index; SSRS, Social Support Rating Scale; PSQI, Pittsburgh Sleep Quality Index.

*p < 0.05, **p < 0.01, ***p < 0.001.

### Multivariable logistic regression model predicting outcomes

The dependent variable was the occurrence of anxiety. Residential area, marital status, education, income, anxiety, social support, sleep quality, and coping style were the independent variables. After multivariable logistic regression, as shown in Table 2, according to the multivariable backward regression analysis, marital status [odds ratio (OR), 5.06; 95% confidence interval (CI), 1.51–17.00; *p* = 0.009], education (OR, 3.16; 95% CI, 1.27–7.88; *p* = 0.036), SSRS score (OR, 1.13; 95% CI, 1.04–1.22; *p* = 0.043), Coping Questionnaire score (OR, 3.36; 95% CI, 1.44–7.80; *p* = 0.002), and HADS anxiety score (OR, 0.09; 95% CI, 0.04–0.19; *p* < 0.001) were significant predictors of depression.

**TABLE 2:** Multivariable logistic regression model predicting outcomes.

Predictors	OR	95% CI	SE	*P*-value
Marital status	5.06	1.51–17.00	0.06	0.009**
Education	3.16	1.27–7.88	0.05	0.036*
SSRS	1.13	1.04–1.22	0.01	0.043*
Coping	3.36	1.44–7.80	0.05	0.002*
Anxiety	0.09	0.04–0.19	0.05	<0.001***

CI, confidence interval; OR, odds ratio; SE, standard error.

*p < 0.05, **p < 0.01, ***p < 0.001.

### Development and application of the depression prediction model

**FIGURE 1:**
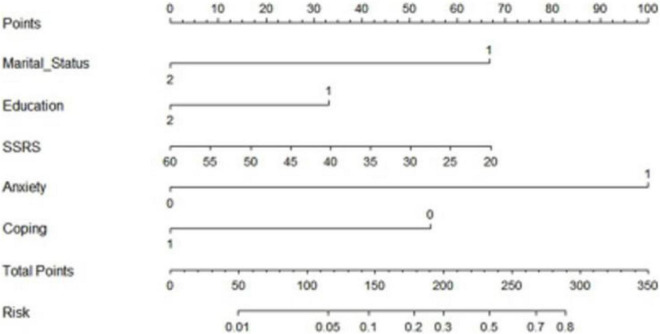
Construction of the depression nomogram.

**FIGURE 2:**
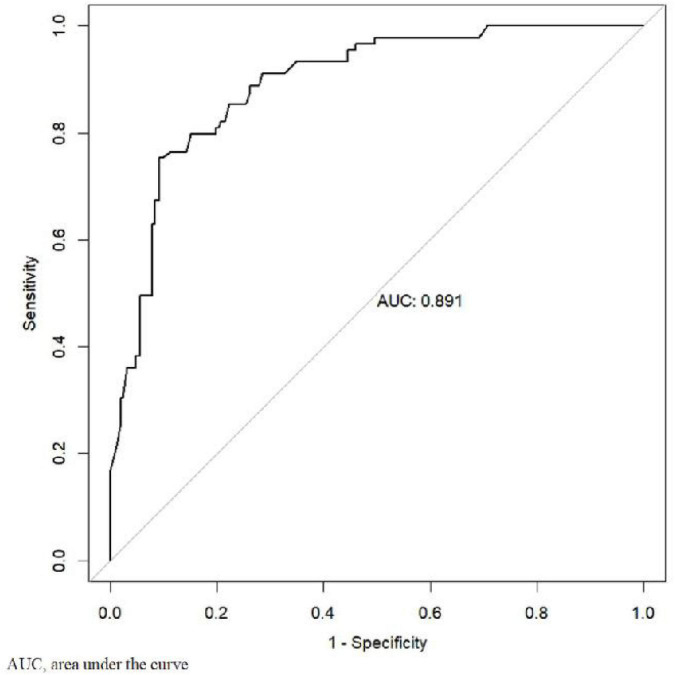
Receiver operating characteristic analyses for prediction of depression.

**FIGURE 3:**
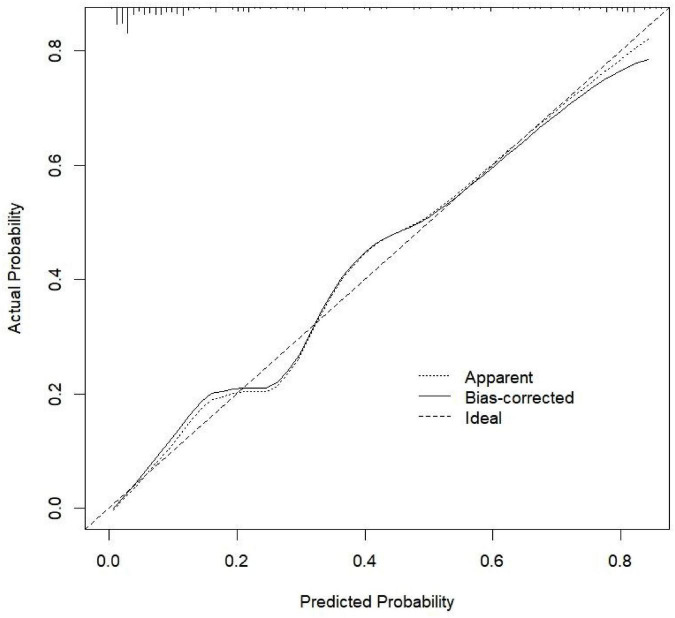
Calibration curve of the nomogram prediction model.

To establish a predictive model for early prevention and intervention, we defined the cutoff for depression as a HADS score >8. To use the nomogram, a response was selected for each variable, with a straight line subsequently drawn to the corresponding point at the top. The total points for all variables were calculated after which the value on the line labeled “total points” was identified. Last, a straight line was drawn from the value on the total point line to the bottom line to determine the risk of depression in patients with SLE (Figure 1). Based on the analysis of the nomogram, the risk of depression was 0.01–0.80 and the area under the receiver operating characteristic curve was 0.891 (Figure 2). The calibration curve can intuitively show that the probability of depression predicted by the nomogram model is consistent with the actual comparison (Figure 3).

## Discussion

The results showed that of the 341 patients with SLE, 26.1% had depression, which was consistent with previous studies (Zhang et al., 2017). Research on depression risk has mainly relied on logistic regression analysis; however, this approach by itself does not allow for the visualization of the risk assessment model. Therefore, we utilized nomogram analysis because it facilitated the development of a visual statistical prediction model for the risk of depression in patients with SLE.

According to recent evidence, lower educational levels among patients with SLE are correlated with increased depressive symptoms (McCormick et al., 2018). In a previous study, lower educational attainment was the demographic predictor most strongly associated with major depression (Knight et al., 2018). It may be that low educational level results in patients being less competitive in their work. These patients also have limited opportunities available to them. This could be possible, as greater educational attainment has been associated with better social functioning and coping skills (Ladin, 2008). Marital status also affects the risk of depression in patients with SLE (Shen et al., 2013) as evidenced by both longitudinal and cross-sectional studies. The quality of the marital relationship may also be significant with regard to depression. Thus, there is a need to pay greater attention to patients’ educational levels and marital status.

Contrary to our findings, a previous study reported a significant correlation between disease activity and depression severity (Alsowaida et al., 2018). Patients with high SLEDAI scores (>12) had an obviously higher rate of depression (40%) compared to the 20% among those with mild or moderate disease (Abd-Alrasool et al., 2017). There was no important or statistically significant difference in the median SLEDAI score between depression severity categories (*p* > 0.05) (Abd-Alrasool et al., 2017). In our study, the mean SLEDAI score was <5 points, indicating low disease activity. The predictive role of SLEDAI scores in depression needs further exploration.

Anxiety usually coexists with depression (Moustafa et al., 2020), and in our study, the nomogram demonstrated that anxiety had a great influence on depression. Given the importance of anxiety and mood disorders and symptoms for overall quality of life, functionality, and health-related outcomes in SLE, the effective management of these conditions is of paramount importance (Papadaki et al., 2019).

Social support refers to the experience of being valued, respected, cared about, and loved by the people in one’s life. It may come from different sources such as family, friends, teachers, community, or any social groups to which one is affiliated (Piel et al., 2017). A healthy marital relationship is a comprehensive source of social support. Previous research shows that low social support is one of the predictors of psychological issues such as depression and anxiety (Roohafza et al., 2014; Galán et al., 2020). Social support has been reported to play an important role in daily life (Roohafza et al., 2014). Support from others eases the pressures of life and reduces depression. In China, family is the most important source of social support. Therefore, we recommend that family members collaborate with healthcare providers in managing patients’ depression.

Coping styles are cognitive efforts to manage stress (Diamond et al., 2006). Similar to social support, coping could play a buffering role with regard to psychological problems. Coping styles can be categorized as active and passive. The former refers to taking a direct and rational approach to deal with a problem, while the latter involves avoidance, withdrawal, and denial (Jia et al., 2004). Active coping styles and perceived social support, particularly positive reinterpretation and support from family, are protective factors for depression and anxiety.

It is well known that depression is a significant contributor to sleep quality disturbances in patients with SLE (Palagini et al., 2014). Moreover, this relationship has been shown in epidemiological studies of sleep in the general population, and with other patient groups (e.g., arthritis, chronic pain) (Katz et al., 2016; Nijs et al., 2018). Vina et al. (2013) reported that depression was related to sleep disturbance, adequacy, and quantity. However, in this study, sleep and depression were related, and the total score of PSQI could not be included in the prediction model, indicating that compared with sleep, other factors have a greater impact.

This is the first published nomogram for depression in patients with SLE. This study explored potential risk factors to develop a model, which was used to predict the risk of depression within one month. Furthermore, the nomogram showed satisfactory validity, discrimination, and clinical utility, indicating good performance, with a high area under the curve of 0.891. A substantial amount of evidence shows that identifying the possibility of depression in patients with SLE in advance and intervening as soon as possible can improve their quality of life (Bachen et al., 2009; Conceição et al., 2019; Margiotta et al., 2019). Nomograms are not only associative but also predictive; the criterion for the inclusion of a variable is its ability to demonstrably improve the probability that the model will correctly predict depression (as quantified by the area under the curve). The establishment of the prediction model provides a tool for the early identification of depression. It also provides a basis for formulating relevant intervention strategies. This nomogram could be used as a convenient screening tool in clinical practice to guide follow-up and aid accurate prognostic assessment. It also provides new ideas for improving the quality of life in patients with SLE.

### Limitations

The present study has several limitations. First, as it was conducted at a single hospital in the Nantong area, the results are not widely generalizable. The next step is to conduct a multi-region survey to ensure that the results are more representative. Second, there is a need to add relevant biological and drug-related indicators to the prediction model.

## Conclusion

This study revealed that marital status, education, social support, coping, and anxiety are predictors for depression in patients with SLE. Moreover, the risk nomogram based on these five factors accurately predicted the likelihood of depression in patients with SLE, which could help clinicians recognize high-risk patients to some extent. Overall, this study highlights the clinical significance of nomograms and the need to use them to screen for depressive symptoms before diagnosis or intervention by doctors.

## Data availability statement

The raw data supporting the conclusions of this article will be made available by the authors, without undue reservation.

## Ethics statement

The studies involving human participants were reviewed and approved by The Ethics Committee of Nantong First People’s Hospital (2017-016). The patients/participants provided their written informed consent to participate in this study.

## Author contributions

HyC and HmC performed the experiment, prepared the figures and the manuscript, and analyzed the data. YG, HyC, TJ, SS, YL, and XC collected the data. BS designed and supervised the experiments and finalized the manuscript. All the authors have read and approved the manuscript.
